# Molecular genetic positioning of small intestine and papilla of Vater carcinomas including clinicopathological classification

**DOI:** 10.1002/cam4.5877

**Published:** 2023-03-31

**Authors:** Masanori Nakamura, Yukiyasu Okamura, Keiichi Ohshima, Teiichi Sugiura, Ryo Ashida, Katsuhisa Ohgi, Etsuro Bando, Keiichi Fujiya, Akio Shiomi, Hiroyasu Kagawa, Taisuke Imamura, Goro Nakayama, Yasuhiro Kodera, Katsuhiko Uesaka, Nobuyuki Ohike, Tomoko Norose, Keiko Sasaki, Takashi Sugino, Sumiko Ohnami, Takeshi Nagashima, Kenichi Urakami, Yasuto Akiyama, Ken Yamaguchi

**Affiliations:** ^1^ Division of Hepato‐Biliary‐Pancreatic Surgery Shizuoka Cancer Center Shizuoka Japan; ^2^ Department of Gastroenterological Surgery Nagoya University Graduate School of Medicine Nagoya Japan; ^3^ Division of Digestive Surgery, Department of Surgery Nihon University School of Medicine Tokyo Japan; ^4^ Medical Genetics Division Shizuoka Cancer Center Research Institute Shizuoka Japan; ^5^ Division of Gastric Surgery Shizuoka Cancer Center Shizuoka Japan; ^6^ Division of Colon and Rectal Surgery Shizuoka Cancer Center Shizuoka Japan; ^7^ Division of Pathology Shizuoka Cancer Center Shizuoka Japan; ^8^ Cancer Diagnostics Research Division Shizuoka Cancer Center Research Institute Shizuoka Japan; ^9^ SRL, Inc. Tokyo Japan; ^10^ Immunotherapy Division Shizuoka Cancer Center Research Institute Shizuoka Japan; ^11^ Shizuoka Cancer Center Hospital and Research Institute Shizuoka Japan

**Keywords:** gene expression, molecular genetics, pancreatic invasion, papilla of Vater carcinoma, small intestine carcinoma

## Abstract

**Background:**

Small intestine carcinoma (SIC) cases in Japan have recently been treated with chemotherapy according to colorectal carcinoma classification, while papilla of Vater carcinoma (PVC) cases according to cholangiocarcinoma (CHC) classification. However, few research reports support the molecular genetic validity of these therapeutic choices.

**Patients and methods:**

Here, we investigated the clinicopathological and molecular genetic factors of SIC and PVC. We used the data from the Japanese version of The Cancer Genome Atlas. Additionally, molecular genetic data on gastric adenocarcinoma (GAD), colorectal adenocarcinoma (CRAD), pancreatic ductal adenocarcinoma (PDAC), and CHC were also referred to.

**Results:**

This study consisted of tumor samples from 12 patients of SIC and three patients of PVC treated from January 2014 to March 2019. Among them, six patients had pancreatic invasion. *t*‐Distributed stochastic neighbor embedding analysis showed that the gene expression pattern of SIC was similar not only to those of GAD and CRAD, but also to that of PDAC in the pancreatic invasion patients. In addition, PVC resembled the GAD, CRAD, and PDAC, rather than the CHC. The molecular genetic characteristics of the six patients with pancreatic invasion were: one had high microsatellite instability, two had a *TP53* driver mutation, and three had tumor mutation burden values <1 mutation/Mb with no driver mutation.

**Conclusions:**

In this study, the extensive gene expression profiling of organ carcinomas newly suggests that SIC or PVC may resemble GAD, CRAD, and PDAC. In addition, the data demonstrate that pancreatic invasive patients may be classified into several subtypes using molecular genetic factors.

## Graphical abstract

Given that the molecular genetic positioning of rare small intestine and papilla of Vater carcinoma were not yet clear, these therapies are still in the exploratory phase. In this study, the molecular genetic characteristics of these carcinomas suggest that they are similar to gastric adenocarcinoma, colorectal adenocarcinoma, and pancreatic ductal adenocarcinoma. Furthermore, it was suggested that pancreatic invasive cases could be classified into several subtypes based on microsatellite instability and driver gene backgrounds, as well as gene expression patterns.

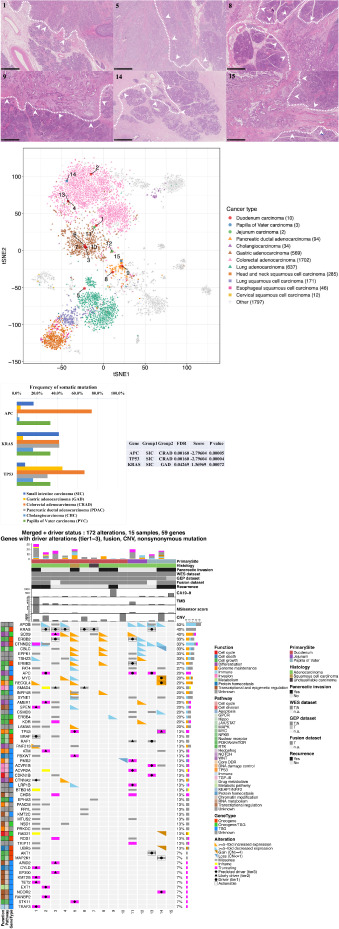

## 1 INTRODUCTION

Small intestine carcinoma (SIC) and papilla of Vater carcinoma (PVC) are adjacent organ carcinomas, but the chemotherapy approaches for treating each are different. SIC, including duodenal carcinoma, is anatomically similar to the adjacent stomach or colon, although it is clinically treated with the same chemotherapy regimen as colorectal carcinoma.^1^ Several studies have reported that the molecular genetic characteristics of SIC are similar to those of gastric and colorectal carcinoma in the adjacent gastrointestinal tract.^2^, ^3^, ^4^ While studies have been performed to classify gastric and colorectal carcinoma into several subtypes based on molecular genetics in addition to clinical pathology findings,^5^, ^6^ there are no such reports yet for SIC due to its rarity.^1^ Furthermore, there have been several reports of genomic analysis using formalin‐fixed, paraffin‐embedded (FFPE) samples in comparison with adjacent organ carcinomas.^7^, ^8^ However, few studies have reported genomic analysis, including gene expression analysis, using fresh‐frozen samples.^2^, ^3^, ^9^

PVC was included as a SIC in the 4th edition of the World Health Organization (WHO) classification of tumors of the digestive system.^10^ It was reclassified as an independent disease from small intestine, gastric, colorectal, and bile duct carcinomas since 2019 in the 5th edition, similar to the Union for International Cancer Control (UICC) classification.^11^, ^12^ However, after several chemotherapy clinical trials worldwide, PVC is often treated as biliary tract cancer.^13^, ^14^ In Japan, PVC is also classified and treated with chemotherapy like bile duct carcinoma.^15^ However, there are few reports on the validity of classifying it as bile duct carcinoma on the molecular genetic level.^16^

In addition, both SIC and PVC are also adjacent to the pancreas. It is known that when tumors invade the pancreatic parenchyma, patient prognosis is poor, although there are also no reported molecular genetic studies on such situations.^17^, ^18^

In this study, multi‐omics analysis was performed by collecting fresh‐frozen samples from various carcinomas of multiple organs.^19^ From these results, SIC and PVC were examined from a comprehensive molecular genetics perspective, including clinicopathological classification, by comparing them with adjacent organ carcinomas. SIC and PVC were also evaluated using available molecular genetic data that especially focused on cases with pancreatic invasion.

## 2 METHODS

### 2.1 Ethics statement

Shizuoka Cancer Center started Project HOPE (High‐Tech Omics‐Based Patient Evaluation) in 2014 to investigate the biological characteristics of cancer and the pathogenesis of individual cases following approval by the Institutional Review Board of Shizuoka Cancer Center (Approval Number 25‐33).^19^ In this project, multi‐omics‐based analyses integrating genomics, transcriptomics, proteomics, and metabolomics were conducted for various types of cancers to develop precision cancer medicine. Project HOPE was designed according to the ethical guidelines for human genome and genetic analysis research, which was revised in 2013.^19^ Written informed consent was always obtained from patients participating in Project HOPE.

### 2.2 Patient selection and study design

Candidates for project HOPE included 5143 Japanese patients who had undergone cancer resection surgery at Shizuoka Cancer Center Hospital. Cancer tissue obtained from these patients helped to establish the Japanese version of The Cancer Genome Atlas (JCGA). This study included tumor samples obtained from SIC and PVC cases that were treated between January 2014 and March 2019, from which data were analyzed for clinicopathological and molecular genetic factors.

Histopathological diagnosis was assessed by at least two pathologists. As the criteria for the histopathological diagnosis of invasion into the pancreas from gastrointestinal cancer, the presence of carcinoma on the mucosa of duodenum or papilla of Vater was observed, without intraepithelial carcinoma of the pancreatic duct. If this could not be determined, we judged the cancer from gastrointestinal tract with the findings that the central of tumor was localized on the mucosal side of the gastrointestinal tract rather than on the pancreatic parenchyma, with invading the pancreatic parenchyma in a continuous manner with the center of tumor. The UICC 8th edition was referenced for staging.^12^

We performed whole exome sequencing (WES) and deep sequencing of the custom cancer panel (CCP) using blood samples collected during the surgery and fresh surgical samples obtained after surgery. Tumor and matched normal samples of SIC or PVC were collected from the luminal side of the gastrointestinal tract. Driver gene mutations were classified as in previous studies, according to predictability: Tier 1 was a driver mutation, Tier 2 was a likely driver mutation, and Tier 3 was a predicted driver mutation.^20^ We defined actionable mutation as genetic mutation with candidate drugs that are expected to have therapeutic effects. Actionable mutations of the target genes for treatment among driver gene mutations were also evaluated. Microsatellite instability (MSI) status was evaluated using MSIsensor.^21^ Samples that met one or more of the following conditions were considered MSI‐High: (1) MSIsensor score of 1 or higher and (2) tumor mutation burden (TMB) ≥10 and the sum of the contributions of the seven mutation signatures associated with MSI ≥0.4. For MSI‐High cases, immunostaining was performed to evaluate the functional loss of mismatch repair protein. We then conducted gene expression profiling (GEP) using matched tumor and adjacent normal tissues from each patient with DNA microarray analysis.^22^, ^23^, ^24^, ^25^ Measurements of WES/CCP and GEP were performed using the Ion Proton system and Agilent system. Experimental procedures are described in detail in previous reports.^20^, ^22^, ^23^ The mean depth of coverage of the target regions was 134.9‐fold for WES and 1173.7‐fold for CCP. The GEP change was considered significant with a fold change of 5 or more or a decrease of −5 or less. A copy number variant (CNV) increase of 4 or more or decrease of less than 1 was considered significant. Molecular genetic data from 574 gastric adenocarcinomas (GAD), 1719 colorectal adenocarcinomas (CRAD), 95 pancreatic ductal adenocarcinomas (PDAC), and 37 cholangiocarcinomas (CHC) from the HOPE study were used for comparison with SIC and PVC.

### 2.3 Statistical analysis

Continuous variables are expressed as median and interquartile range (IQR) and were compared using the Mann–Whitney *U*‐test. Statistical analysis was performed using EZR (Saitama Medical Center, Jichi Medical School), a graphical user interface for R (The R Foundation for Statistical Computing). *p* < 0.05 was considered significant.^26^ For the statistical analysis of adjacent organ carcinoma comparisons, the ratio between the two groups was tested by Fisher's exact establishment test and corrected for multiple testing by the Benjamini–Hochberg method. A false discovery rate (FDR) <0.05 was considered statistically significant. The score value was calculated by [Score = −log10 (FDR)], where a larger value indicates a more significant difference; the value multiplied by −1 was listed for those with a higher frequency in Group 2 compared with those with a higher frequency in Group 1. Thus, a large positive score value indicated a significantly higher frequency of Group 1 compared with that of Group 2, while a large negative score value indicated a significantly higher frequency of Group 2 compared with that of Group 1. *t*‐Distributed stochastic neighbor embedding (*t*‐SNE) analysis was performed using the GEP dataset from JCGA with the “Rtsne” package (RRID:SCR_016900, https://github.com/jkrijthe/Rtsne).^20^

## 3 RESULTS

### 3.1 Clinicopathological data

**FIGURE 1:**
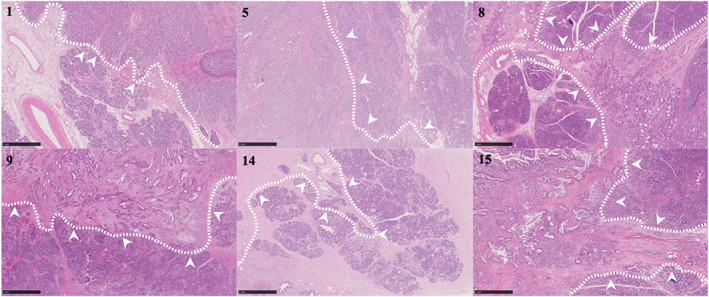
Histopathological examination findings. The pictures of histopathological examination of six cases with pancreatic infiltration are shown. The number shown is the case number. The extent of the tumor is indicated by white dotted lines. The site of invasion is noted by arrowheads. The side with the arrowheads is the normal pancreatic tissue side from the white dotted line. The length of the black bar is equivalent to 1 mm scale.

A summary of the patients' data is shown in Table 1. The median age was 71 years (IQR: 64–74). A total of 15 patients were sampled, including 12 SIC patients and three PVC patients. The 12 individuals with SIC consisted of 10 duodenal carcinoma patients and two jejunal carcinoma patients. Histopathologically, 13 were classified as adenocarcinoma (86.6%), making it the most common among the 15 patients. The remaining two patients were classified as squamous cell carcinoma in one patient (6.6%) and epithelial unclassifiable origin carcinoma in one patient (6.6%). Patients with small tumors tended to be excluded from Project HOPE because of the difficulty of pathological diagnosis when tumor tissue samples were removed. The median size of the tumors was 39 mm (range: 18–97). There were seven patients with invasion of other organs, six of which had pancreatic invasion (Figure 1, Case Nos. 1, 5, 8, 9, 14, and 15). Among the six patients with pancreatic invasion, four patients had postoperative recurrence (Table 1). Of the nine patients without pancreatic invasion, only one patient suffered from recurrence.

**TABLE 1:** Clinicopathological characteristics of this study. The case number indicates the sample for each case.

Case	Primary site	TMB	Histology	Stage (UICC)	Invasion of other organs	Overall survival (months)	Recurrence	Prognosis
1	Duodenum	39.884	Adenocarcinoma	IIIA (T4N1M0)	Pancreas, colon	3	Present	Death
2	Duodenum	6.767	Adenocarcinoma	IIB (T4N0M0)	Colon	7	Present	Unknown
3	Duodenum	6.608	Adenocarcinoma	IA (T3N0M0)		23	Absent	Alive
4	Duodenum	3.237	Adenocarcinoma	I (T1N0M0)		28	Absent	Alive
5	Duodenum	1.891	Squamous cell carcinoma	IIIA (T4N1M0)	Pancreas	60	Absent	Alive
6	Duodenum	0.832	Adenocarcinoma	IIIB (T4N2M0)		37	Absent	Death
7	Duodenum	0.712	Unclassifiable carcinoma	IIIA (T3N1M0)		29	Absent	Alive
8	Duodenum	0.118	Adenocarcinoma	IIB (T4N0M0)	Pancreas, common bile duct	42	Absent	Alive
9	Duodenum	0.093	Adenocarcinoma	IIIB (T4N2M0)	Pancreas, common bile duct	7	Present	Unknown
10	Duodenum	0.061	Adenocarcinoma	IIIA (T3N1M0)		53	Absent	Alive
11	Jejunum	16.019	Adenocarcinoma	IIB (T4N0M0)		76	Absent	Alive
12	Jejunum	0.266	Adenocarcinoma	IIA (T3N0M0)		39	Absent	Alive
13	Papilla of Vater	3.640	Adenocarcinoma	IB (T1bN0M0)		23	Absent	Alive
14	Papilla of Vater	3.376	Adenocarcinoma	IV (T3bN2M1)	Pancreas	5	Present	Death
15	Papilla of Vater	0.147	Adenocarcinoma	IV (T3aN1M1)	Pancreas	17	Present	Death

Abbreviation: TMB, tumor mutation burden (mutation/MB).

### 3.2 Comprehensive gene expression analysis

**FIGURE 2:**
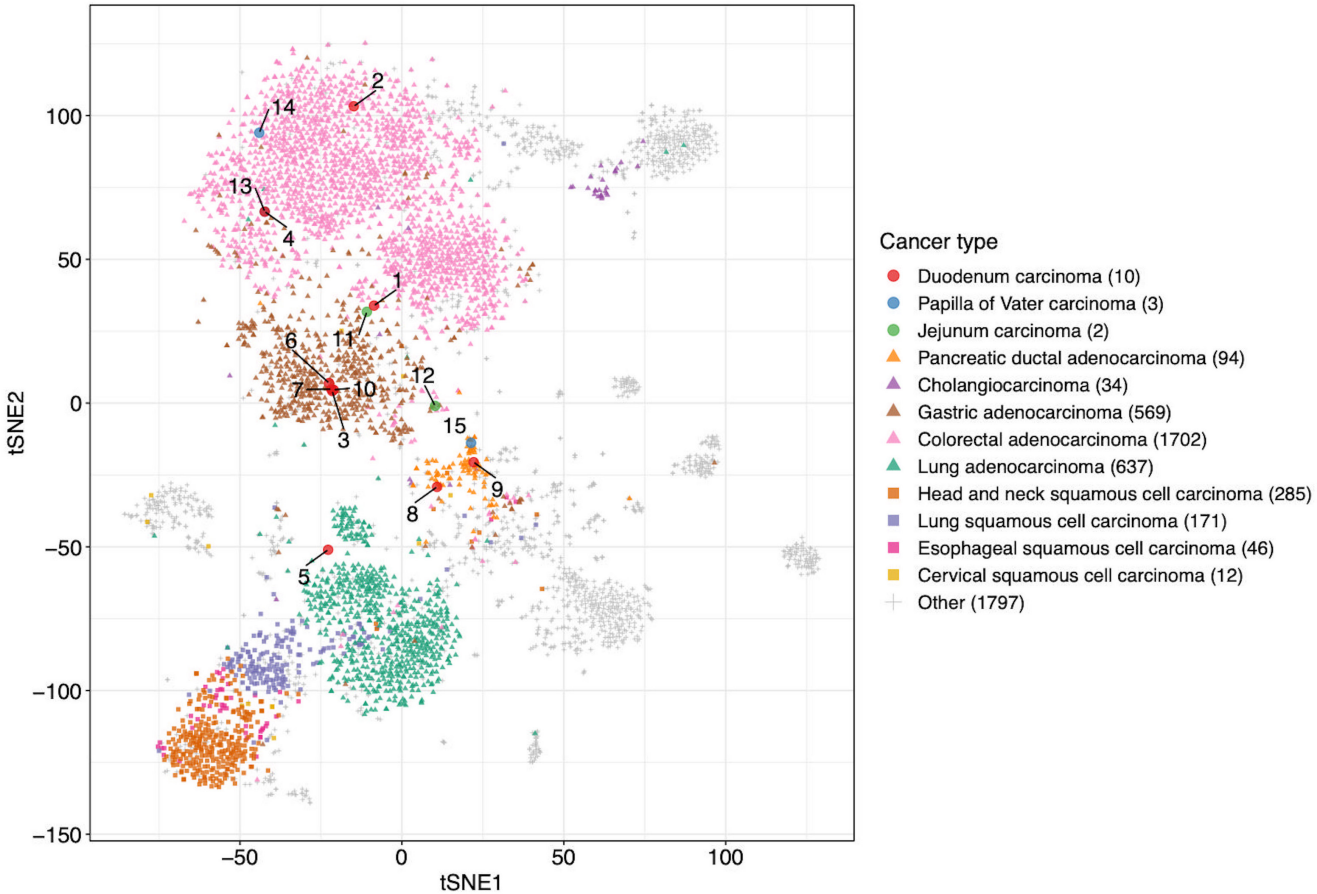
We performed *t*‐distributed stochastic neighbor embedding analysis from all gene expression data of 5143 patients. The cancer types of the cases in this study, such as the duodenum, papilla of Vater, and jejunum, are dotted. The case numbers are the same as in Table 1. Gastric adenocarcinoma, colorectal adenocarcinoma, pancreatic adenocarcinoma, cholangiocarcinoma, lung adenocarcinoma, head and neck squamous cell carcinoma, lung squamous cell carcinoma, esophageal squamous cell carcinoma, cervical squamous cell carcinoma, and other carcinomas are also plotted for comparison. Numbers in parentheses indicate the number of cases of each cancer type.

In our previous study, we evaluated 5521 fresh frozen tumor tissues obtained from 5143 Japanese cancer patients through comprehensive GEP.^20^ Based on the comprehensive GEP data in various tumors, the results of a two‐dimensional *t*‐SNE analysis are shown in Figure 2. This *t*‐SNE plot is distributed along the expression levels of individual carcinoma types. Three (nos. 8, 9, and 15) of the 15 patients showed similar expression patterns to pancreatic cancer. All three patients had histopathological pancreatic invasion; thus, all had a TMB of less than 1 in WES analysis. All gene expression patterns of the 11 patients were similar to those in the GAD or CRAD cluster, except for patients in the PDCA cluster or duodenal squamous cell carcinoma. The duodenal squamous cell carcinoma (no. 5) in this patient was plotted within the cluster of lung adenocarcinoma located between lung squamous cell carcinoma and PDAC. The gene expression pattern of PVC (nos. 13–15) was plotted in clearly different positions from that of CHC.

### 3.3 Molecular genetic comparison of SIC or PVC with adjacent organ carcinomas

**FIGURE 3:**
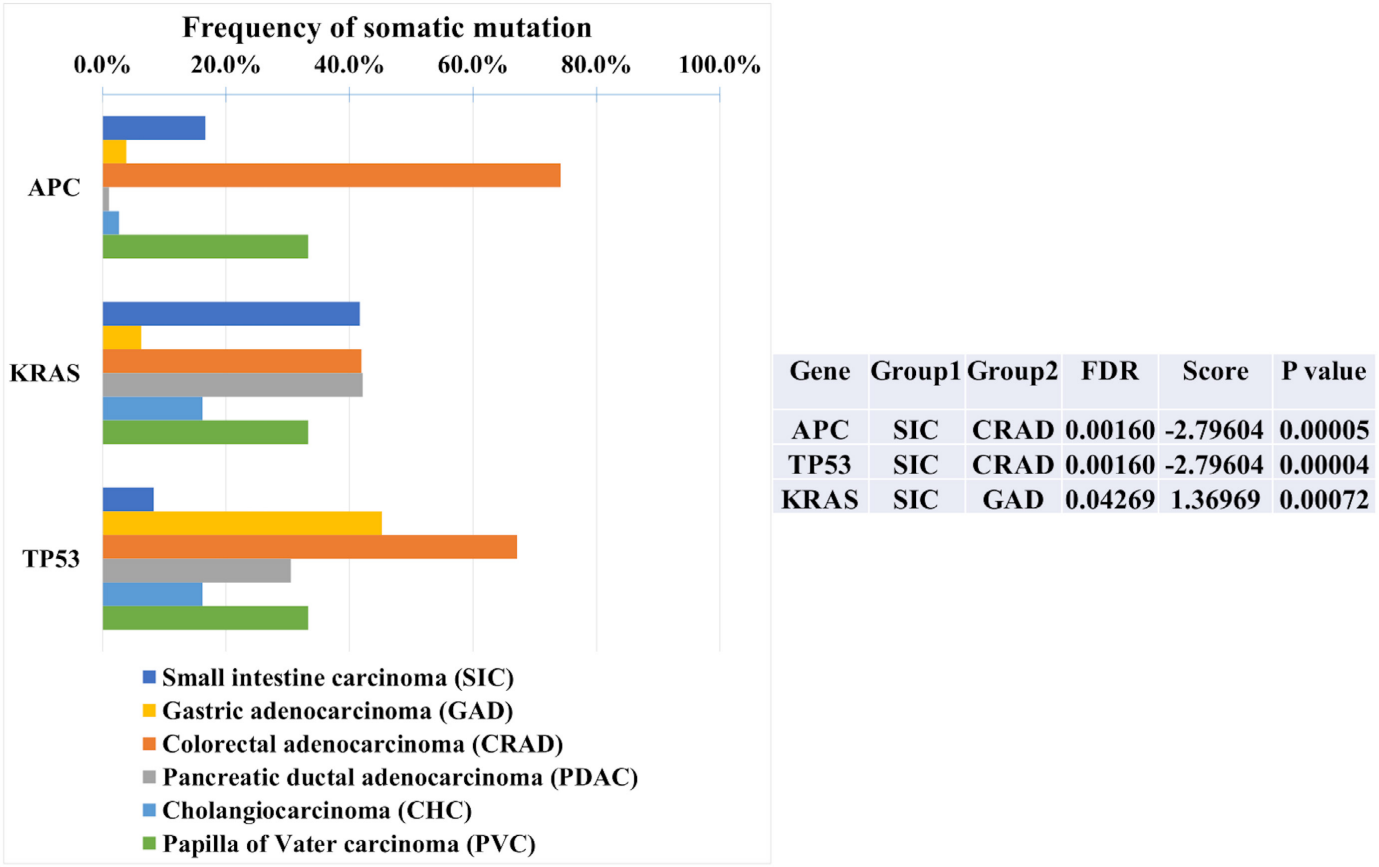
The HOPE study data showed somatic mutations with statistically significant differences in mutation frequency between 12 SIC or three PVC with adjacent organ carcinomas. These included gastric adenocarcinoma (GAD), colorectal adenocarcinoma (CRAD), pancreatic ductal adenocarcinoma (PDAC), and cholangiocarcinoma. *p*‐Values were obtained by comparing Group 1 and Group 2 carcinomas for the corresponding genes using the Fisher exact test. The results corrected for multiple testing were then shown as false discovery rate (FDR). FDR <0.05 was defined as a statistically significant difference. The score value was calculated by [Score = −log10 (FDR)], where higher values indicate greater significant differences. Positive score values indicate that Group 1 is larger, while negative score values indicate that Group 2 is larger. TP53 and APC had significantly lower mutation frequencies in SIC than in CRAD, and KRAS had a significantly higher mutation frequency in SIC than in GAD.

We compared SIC with adjacent organ carcinomas, including GAD, CRAD, PDAC, and CHC, using 59 genes as a Tier 1, 2, or 3 driver gene in this study (Figure 3). TP53 and APC had significantly lower frequencies of mutations in SIC than in CRAD, while KRAS had a significantly higher frequency of mutations in SIC than in GAD. Furthermore, APOB had significantly more frequent decreased gene expression in SIC than in CRAD or PDAC (Figure S1). In contrast, there were no significant differences in CNV among the carcinomas. For pathway mutations (Figure S2), the MAPK pathway was more frequently mutated in SIC than in GAD. The WNT pathway was mutated less frequently in SIC than in CRAD, and more frequently in SIC than in GAD or PDAC. TP53 pathway mutations were less frequent in SIC than in CRAD.

Similar to SIC, we compared PVC with adjacent organ carcinomas of GAD, CRAD, PDAC, and CHC (Figure 3). For gene expression (Figure S1), *APOB* had significantly more frequent decreased gene expression in PVC than in CRAD. There were no significant differences for somatic mutations and CNV, nor any significant differences for pathway mutations (Figure S2).

### 3.4 Somatic mutation overview

**FIGURE 4:**
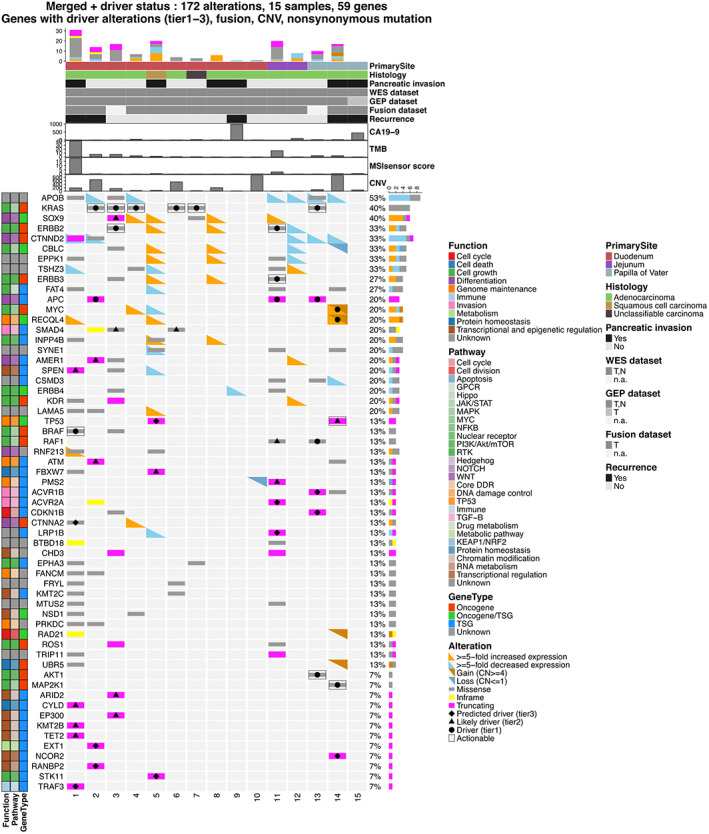
The oncoprint of this study. The mutation signatures and pathway alterations are shown. The primary site, histology, pancreatic invasion, relapse, CA19‐9, tumor mutation burden, MSIsensor score, and copy number variation are shown from top to bottom, respectively. Each row represents a sample.

Based on the WES and CCP of SIC and PVC, the gene mutations are summarized in the order of TMB in duodenal carcinoma, jejunal carcinoma, and PVC (Figure 4). Specific values for the increase or decrease in GEP and CNV for each gene were obtained for each patient (Table S2). The driver gene with the highest frequency of mutations was KRAS (6 of 15 patients; 40%). KRAS mutation was detected in five patients in WES and six patients in CCP, including one patient that was undetected in WES but detected in CCP (no. 7). All five patients in WES had KRAS missense mutations (c.35G>A, p. Gly12Asp; c.35G>C, p. Gly12Ala; c.34G>T, p. Gly12Cys; c.38G>A, p. Gly13Asp; c.182A>T, p. Gln61Leu). Other than a *KRAS* mutation, the driver genes with higher frequencies of mutations were APC in two patients (16%) and *TP53* in one patient (8%) among the 12 SIC patients. No histology‐specific or organ‐specific driver genes were identified. When divided by TMB with 1 as a cutoff value, patients with lower TMB values (TMB <1 mutation/MB) had significantly fewer mutations in Tier 1 driver genes (median 0 vs. 1, *p* = 0.011) and actionable mutations (median 0 vs. 1, *p* = 0.018) compared with patients with higher TMB values (TMB ≥1 mutation/MB).

In this study, two patients (nos. 1 and 11) were determined to be MSI‐High. For these two patients, the expression of MLH1, MSH2, MSH6, and *PMS2* was examined using immunostaining tests. One of these patients had jejunal carcinoma, was already clinically suspected to have Lynch syndrome, and was immunohistochemically confirmed to have a *PMS2* mutation during diagnosis. WES and Sanger sequencing performed in this study confirmed the presence of a germline mutation (p.I18Sfs*34) and a somatic mutation (p.Leu420fs). The second patient had duodenal carcinoma with pancreatic invasion, in which BRAF V600E was found to be a driver gene mutation in the WES and was immunohistochemically confirmed to have MLH1 and PMS2 deletions.

### 3.5 Gene mutation of pancreatic invasion case

Histopathologically, six of the 15 patients showed pancreatic invasion (Table 1, Figures 1, 4). One patient with pancreatic invasion was classified as MSI‐High with high TMB values (≥15 mutation/Mb). In contrast, there were three patients with pancreatic invasion with low TMB values (<1 mutation/Mb) and no driver mutation genes detected (nos. 8, 9, and 15). Additionally, among the patients with pancreatic invasion, there were two with *TP53* as a driver mutation, indicating that they were not MSI‐High and had 15 mutation/Mb>TMB values ≥1 mutation/Mb. These two *TP53* mutations were a Tier 3 driver mutation (c.450_460delACCCCCGCCCG, splice acceptor) in one patient of squamous cell carcinoma of the duodenum (no. 5) and a Tier 2 driver mutation (c. 994‐1G>A, splice acceptor) in one patient of PVC (no. 14). In the PVC patient with *TP53* as the driver gene (no. 14), there was an increase in CNV and MYC, and RECQL4 gene expression levels. KRAS mutations, which were more frequent in the overall study, were not found in any of the pancreatic invasion patients.

## 4 DISCUSSION

Some genome‐related studies have been reported on SIC and PVC, though to the best of our knowledge, few studies have performed the analysis with next generation sequencing on fresh‐frozen samples.^2^, ^3^, ^9^ There has been only one study comparing FFPE samples to adjacent organ carcinomas, which compared GAD and CRAD by molecular genetics.^7^ In this respect, Project HOPE has a big advantage in that it supports comparisons among various types of carcinomas.^19^, ^20^, ^21^, ^22^, ^23^, ^24^, ^25^, ^27^ Moreover, the strong point of this study is that it is an overview study of the whole image which shows the positioning of SIC and PVC in rare cancers among adjacent organ carcinomas, focusing on driver gene mutations, pathway mutations, CNV, and gene expression not only in GAD and CRAD, but also in PDAC and CHC, using fresh‐frozen samples. Among the genetic data obtained from 5143 patients with various carcinomas, it would be valuable for future studies to investigate which carcinomas have gene expression patterns that resemble those of SIC and PVC, which have ambiguous molecular genetic backgrounds because of their rare occurrence.

As a result, while several studies have reported that the molecular biological characteristics of SIC are similar to those of CRAD in the adjacent gastrointestinal tract,^1^, ^4^ the present study showed that the gene expression pattern of SIC was similar not only to that of GAD and CRAD, but also to that of PDAC in the pancreatic invasion patients. Additionally, PVC did not resemble CHC, but rather resembled GAD, CRAD, and PDAC, suggesting it might be appropriate to classify it separately from CHC, as in the UICC or WHO classifications. Considering that in Japan, chemotherapy for SIC has recently been given according to CRAD and for PVC according to CHC, the findings of the current study suggest that chemotherapy with reference to molecular genetic subtypes is an option.

There are several panel sequencing datasets for SIC, but not for PVC to our knowledge.^28^, ^29^, ^30^ In those SIC datasets, TP53, KRAS, APC, PIK3CA, SMAD4, ERBB2, and BRAF are representative gene mutations (Table S3). Our data showed that TP53 and APC had significantly lower frequencies of mutations in SIC than in CRAD, while KRAS had a significantly higher frequency of mutations in SIC than in GAD. For the mutations in key driver genes, the present study showed that the mutations in key driver genes between SIC and CRAD or GAD were different, which is similar to previous reports of FFPE studies.^7^ For gene expression, APOB had significantly more frequent decreased gene expression in SIC than in CRAD and PDAC, and in PVC than in CRAD, which has not been reported previously. Previous studies on fresh‐frozen samples have also reported a higher mutation frequency in the WNT pathway in SIC with respect to pathway mutations, which is certainly higher than in GAD or PDAC.^2^, ^3^ However, the WNT pathway mutation frequency was significantly lower than that of CRAD in the current study, indicating that it was not specific to SIC. Furthermore, the MAPK pathway mutations differed from GAD and TP53 pathway mutations from CRAD in SIC. Interestingly, these results suggest that SIC is unique from GAD and CRAD at the driver gene level, whereas it resembles either GAD, CRAD, or PDAC in overall gene expression.

Our data newly showed that APOB gene expression was significantly decreased in PVC compared with that in CRAD. No statistically significant differences between each carcinoma were observed for somatic mutations, CNV, or pathway mutations. Unfortunately, this result may be from the small number of PVC patients included in this study. Other literature was cited to supplement the discussion. There are two relatively large case‐count surveys in PVC: the study by Yachida et al. using fresh‐frozen samples and the study by Gingras et al. using FFPE samples. In these studies, the association between genetic mutations and histological phenotypic subtypes was analyzed. Their findings suggested that the frequencies of KRAS, TP53, and SMAD4 mutations were increased in the pancreaticobiliary type, while the incidences of APC, TP53, and KRAS mutations were increased in the intestinal type.^16^, ^31^ These phenotypes are reportedly associated with prognosis in SIC.^32^ However, no studies have reported on whether these phenotypes and the adjacent organ carcinomas have molecular genetics similarities. This issue is likely to be addressed in the future.

The frequency of MSI‐High cases in SIC ranges from 5% to 35%, and the proportion in this study (16%) fell within this range.^1^ In CRAD, MSI‐High cases are classified into sporadic cases from somatic mutations and hereditary cases associated with germline mutations.^33^ Similarly, in the present SIC study, patients with the somatic mutation type and Lynch syndrome were also extracted as MSI‐High cases. It is well known that immune checkpoint inhibitors are effective in these MSI‐High cases, which are also characterized by high TMB levels.^34^, ^35^ Both patients with SIC in this study had high TMB levels, so immune checkpoint inhibitors could be an option at the time of recurrence.

In lung cancer, oncological tumors with actionable mutations exist even with low TMB.^36^ In the present study, patients with lower TMB values (TMB <1 mutation/MB) had significantly fewer driver gene mutations than patients with higher TMB values (TMB ≥1 mutation/MB), resulting in fewer actionable mutations in the lower TMB patients. In other words, although treatment targeting actionable mutations can be expected in lung cancer patients with lower TMB levels, the results of this study suggest that immune checkpoint inhibitors are not an option for patients with lower TMB levels of SIC and PVC, and that treatment targeting actionable mutations is unlikely. In the future, it is desirable to identify new therapeutic targets by whole genome sequencing in patients with low TMB values.

Because both SIC and PVC are adjacent to the pancreas and pancreatic invasion is reportedly a poor prognostic factor,^17^, ^18^ the patients with pancreatic invasion in this study were investigated using a molecular genetic approach. Six patients with SIC and PVC with pancreatic invasion showed various patterns of gene expression. Our data suggested that three patients had a PDAC‐like pattern, one patient had a CRAD‐like pattern, one patient had a border between GAD and CRAD, and one patient with squamous cell carcinoma was near clusters of squamous cell carcinomas in other organs. Although the patients showed a variety of patterns, PDAC‐like patients were the most common. It was proposed that either the tumor itself that is causing pancreatic invasion resembles PDAC at the gene expression level or the pancreatic tissue undergoing invasion is the source of the expression pattern. Although the expression levels of driver genes in SIC or PVC patients with pancreatic invasion were not consistent with the genes that are upregulated or downregulated in PDAC, the comprehensive *t*‐SNE analysis oddly showed these patients to be similar to PDAC. This is an issue to be resolved in the future. For gene expression, TMB, MSI, and driver genes, the patients could be classified into three molecular genetic subtypes. The molecular genetic characteristics of the six patients with pancreatic invasion were MSI‐High in one, TP53 driver mutation in two, and TMB <1 mutation/MB without driver mutation (PDAC‐like gene expression) in three. For prognosis, four of the six patients with pancreatic invasion showed recurrence: in one patient with MSI‐High, in one with TP53 mutation, and in two with TMB value <1 (PDAC‐like gene expression). The results of this study suggest that the pancreatic invasion of SIC and PVC, which are considered to have a poor prognosis, may have different molecular genetic backgrounds among them.

There are several limitations of this study. First, the sample size of this study was small because of the rarity of this carcinoma. The anatomic complexity of PVC makes strict classification difficult, and there are few reports of molecular genetic analysis of small intestine and PVC combined. Therefore, it is desirable to clarify the molecular genetics of SIC and PVC in a multicenter study. Second, when excised samples with small tumor diameters were removed for genetic studies, subsequent histopathological diagnosis has been difficult. Therefore, early‐stage carcinomas tended to be excluded from this study. Third, in the case of pancreatic invasion, the duodenum and pancreas are anatomically adjacent to each other, so there is a possibility of PDAC‐derived tissue. However, although KRAS mutations have been reported to be highly prevalent in PDAC,^37^ this possibility is unlikely here because KRAS mutations were not observed in all patients with pancreatic invasion in this study. Fourth, especially for patients with pancreatic invasion, it may be advisable to perform gene mutation and gene expression analyses on FFPE samples to evaluate errors potentially arising from tumor heterogeneity. Unfortunately, that was not possible in this study. It is desirable to evaluate the results when the number of cases is accumulated.

In conclusion, SIC is clinically treated with chemotherapy methods similar to those used for CRAD, and PVC is classified as CHC in Japan. However, a new finding from GEP of extensive organ carcinomas in this study suggests that these carcinomas may be similar to GAD, CRAD, and PDAC. Additionally, it was also newly suggested that pancreatic invasive cases may be classified into several subtypes based on MSI‐High and driver gene backgrounds, as well as gene expression patterns. In SIC and PVC, it is clinically desirable to consider a treatment based not only on anatomical classification but also on molecular genetic profiling. However, this will require not only panel sequences but also WES and GEP data, which will take more time and cost to develop the environment.

## AUTHOR CONTRIBUTIONS

**Masanori Nakamura:** Conceptualization (lead); writing – original draft (lead). **Keiichi Ohshima:** Conceptualization (supporting); supervision (equal). **Teiichi Sugiura:** Data curation (equal); resources (equal). **Ryo Ashida:** Data curation (equal); resources (equal). **Katsuhisa Ohgi:** Data curation (equal); resources (equal). **Etsuro Bando:** Data curation (equal); resources (equal). **Keiichi Fujiya:** Data curation (equal); resources (equal). **Akio Shiomi:** Data curation (equal); resources (equal). **Hiroyasu Kagawa:** Data curation (equal); resources (equal). **Taisuke Imamura:** Conceptualization (supporting); writing – review and editing (supporting). **Goro Nakayama:** Conceptualization (equal); writing – review and editing (supporting). **Yasuhiro Kodera:** Conceptualization (supporting); writing – review and editing (supporting). **Katsuhiko Uesaka:** Data curation (equal); resources (equal). **Nobuyuki Ohike:** Data curation (equal); resources (equal). **Tomoko Norose:** Data curation (equal); resources (equal). **Keiko Sasaki:** Data curation (equal); resources (equal). **Takashi Sugino:** Data curation (equal); resources (equal). **Sumiko Ohnami:** Data curation (equal); resources (equal). **Takeshi Nagashima:** Formal analysis (equal); visualization (equal). **Kenichi Urakami:** Conceptualization (equal); writing – review and editing (supporting). **Yasuto Akiyama:** Project administration (equal); supervision (equal). **Ken Yamaguchi:** Project administration (equal); supervision (lead).

## CONFLICT OF INTEREST STATEMENT

The authors have no conflict of interest to declare.

## ETHICS STATEMENT

Shizuoka Cancer Center started Project HOPE (High‐Tech Omics‐Based Patient Evaluation) in 2014 to investigate the biological characteristics of cancer and the pathogenesis of individual cases following approval by the Institutional Review Board of Shizuoka Cancer Center (Approval Number 25‐33).

## Supporting information


Figure S1.

Figure S2.

Table S1.

Table S2.

Table S3.

## Data Availability

The data that support the findings of this study are available on request from the corresponding author. The data are not publicly available due to privacy or ethical restrictions.
