# Supplementary figures and images for: Molecular genetic positioning of small intestine and papilla of Vater carcinomas including clinicopathological classification
Source: Cancer Med. 2023 Mar 31;12(10):11491–502. doi: 10.1002/cam4.5877 (PMC10242328; doi:10.1002/cam4.5877)

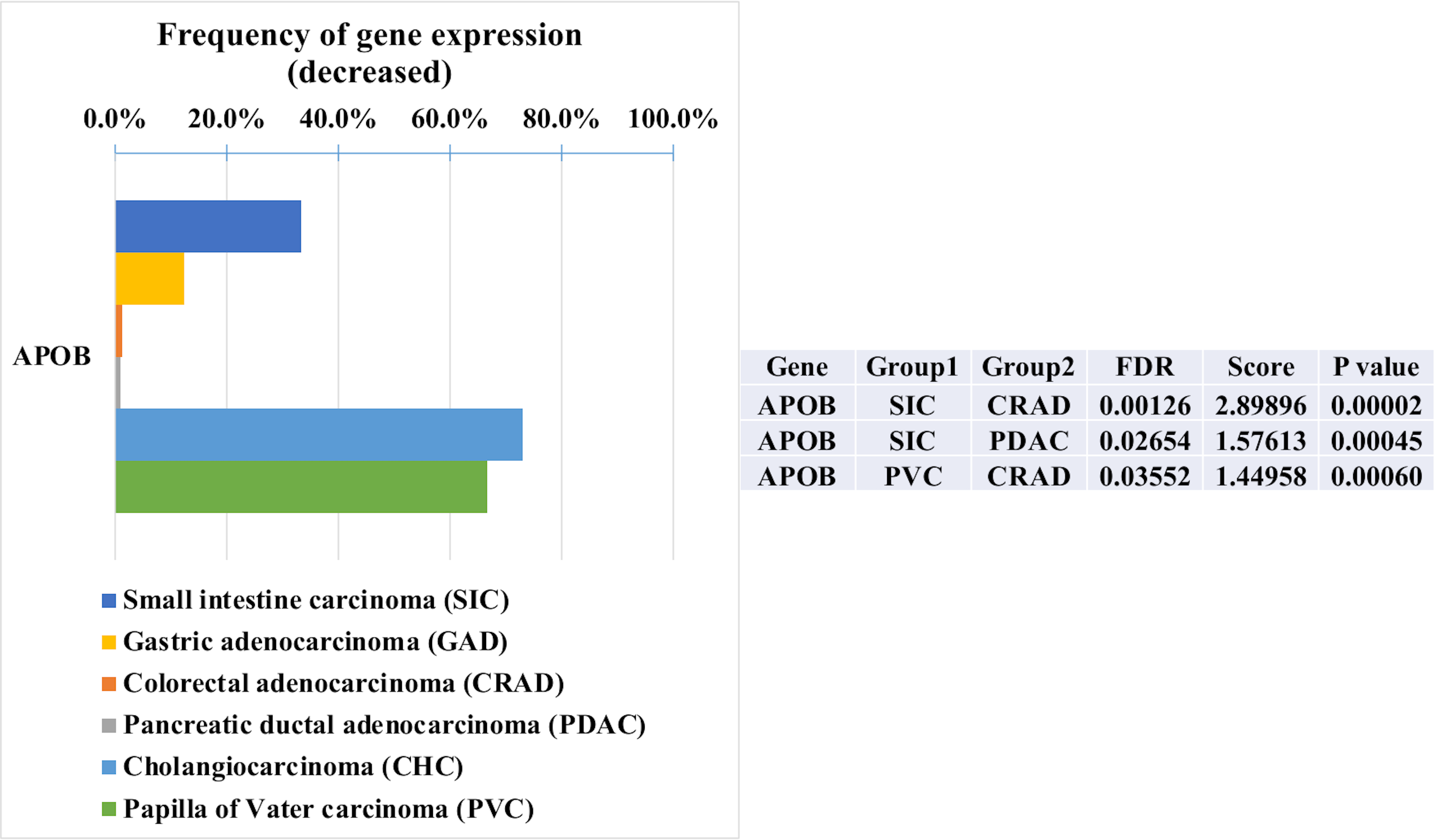

Supplement: Supplementary file 1 — Figure S1. [file CAM4-12-11491-s005.tiff]

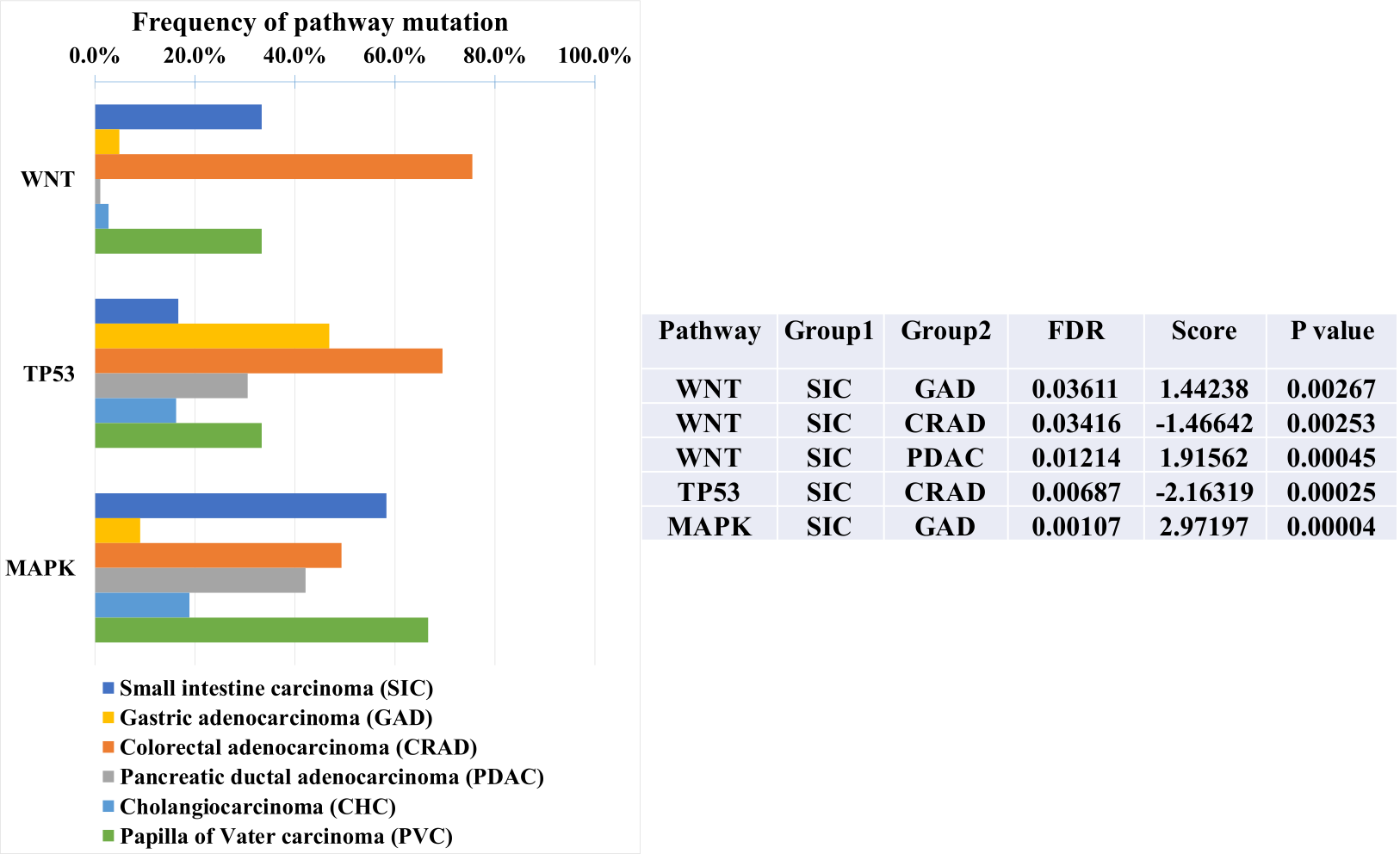

Supplement: Supplementary file 2 — Figure S2. [file CAM4-12-11491-s004.tiff]
